# Impact of COVID-19 lockdown on glycemic levels during pregnancy: A retrospective analysis

**DOI:** 10.1515/med-2023-0862

**Published:** 2023-12-07

**Authors:** Erika Di Zazzo, Sergio Davinelli, Serena Panichella, Giovanni Scapagnini, Mariano Intrieri, Silvio Garofalo

**Affiliations:** UOC Laboratorio Analisi, Ospedale “A. Cardarelli”, 86100 Campobasso, Italy; Department of Medicine and Health Sciences “V. Tiberio”, University of Molise, 86100 Campobasso, Italy

**Keywords:** COVID-19, lockdown, gestational diabetes mellitus, pregnancy, glycemia, oral glucose tolerance test

## Abstract

Studies on the COVID-19 pandemic effects on gestational diabetes mellitus (GDM) remain limited and controversial. This study aimed to investigate the impact of the COVID-19 lockdown on the glycemic balance of pregnant women and GDM risk. To this aim, a single-center retrospective cohort analysis assessing glucose homeostasis using the oral glucose tolerance test in 862 pregnant women before (from March 9, 2019 to March 8, 2020 – Group 1), during (from March 9, 2020 to March 8, 2021 – Group 2), and after (from March 9, 2021 to March 8, 2022 – Group 3) the COVID-19 lockdown in Molise, a region of central Italy, was conducted. We observed that the blood glucose concentration of pregnant women was significantly lower during the COVID-19 lockdown than during the previous and following years at all time points evaluated (time 0, 60′, and 120′). Specifically, at time 0, it was 82.14 mg/dl for group 2 vs 85.94 for group 1 (*p* = 0.0001) and 85.87 for group 3 (*p* = 0.001). Similarly, at 60′, it was 121.38 mg/dl for group 2 vs 129.30 mg/dl for group 1 (*p* = 0.0029) and 131.68 mg/dl for group 3 (*p* = 0.0006). Moreover, at 120′, it was 104.20 mg/dl for group 2 vs 111.51 mg/dl (*p* = 0.0004) for group 1, and 116.06 mg/dl for group 3 (*p* = 0.0001). In contrast with previous findings, the COVID-19 lockdown was associated with an improved glycemic balance. Further studies are needed to better clarify the influence of lockdown restrictions on glucose metabolism and, consequently, on GDM risk.

## 1 Introduction

Gestational diabetes mellitus (GDM) is defined as glucose intolerance of variable degree with onset or first recognition during pregnancy and represents one of the most common pregnancy complications [1]. Depending on the population features and diagnostic criteria, the GDM prevalence varies from 5.8% in Europe to 12.9% in the Middle East and North Africa. GDM is associated with adverse pregnancy outcomes and long-term maternal complications, including premature delivery, cesarean delivery, preeclampsia, type 2 diabetes (T2D), and cardiovascular diseases [2,3]. Likewise, prenatal exposure to maternal hyperglycemia may lead to fetal hyperinsulinemia, increasing the risk of macrosomia, neonatal hypoglycemia, hyperbilirubinemia, and adult T2D [4]. Therefore, maintenance of physiological blood glucose concentrations during pregnancy may reduce maternal and fetal morbidity [5].

The oral glucose tolerance test (OGTT) is considered the cornerstone of GDM diagnosis. Although OGTT is usually conducted between 24 and 28 weeks of gestation, high-risk pregnancies, with a history of GDM, are tested between 16 and 18 weeks [6]. Several studies have demonstrated the relevance of an appropriate identification and management of pregnant women with GDM. Prospective human studies have also demonstrated that exposure to stressful life events may cause alterations in glucose metabolism and lead to metabolic syndrome, prediabetes, or undetected T2D [7,8,9]. In the context of the Coronavirus disease 2019 (COVID-19) pandemic, it has been reported that both clinicians and pregnant women are unwilling to recommend or undergo OGTT [10]. Moreover, preliminary data show that social distancing, lockdown, and home confinement during the COVID-19 pandemic caused changes in dietary habits and a reduction in physical activity levels among pregnant women, leading to uncontrolled glycemia [11,12].

Although studies on the effects of the COVID-19 pandemic on pregnant women with or without GDM remain limited, it has been noted that diabetes control was lower during the COVID-19 pandemic lockdown, especially in women with GDM [13]. The stressful lockdown phase also influenced gestational weight gain, which was higher during the lockdown months [14]. A recent analysis revealed a significant increase in GDM prevalence in women who were pregnant during the COVID-19 pandemic compared to the matched group of women who gave birth in 2019 [15]. Here, we conducted a retrospective analysis to compare the glycemic balance in pregnant women before, during, and after the COVID-19 lockdown in Italy.

## 2 Methods

### 2.1 Study design

This retrospective single-center study was conducted in the U.O.C. Laboratory of “Cardarelli” Hospital, Campobasso, Italy. We analyzed blood samples collected from 862 pregnant (mostly caucasian) women who participated in a screening test for GDM. All participants aged between 17 and 47 years were tested between March 9, 2019 and March 8, 2022. Women were subdivided into groups based on three-time intervals as follows: 435 pregnant women before the COVID-19 lockdown (from March 9, 2019 to March 8, 2020 – Group 1), 193 pregnant women during the COVID-19 lockdown (from March 9, 2020 to March 8, 2021 – Group 2), and 234 pregnant women after the COVID-19 lockdown period (from March 9, 2021 to March 8, 2022 – Group 3). According to the current literature and national guidelines [16,17,18,19], screening for GDM diagnosis is performed by OGTT, evaluating glycemia at time 0 and at 1 and 2 h after the administration of a solution containing 75 g glucose [20]. GDM was diagnosed when a single OGTT value reached or exceeded the cutoff (fasting blood glucose, 92 mg/dl; 1 h OGTT value, 180 mg/dl; or 2 h OGTT value, 153 mg/dl). Women with pregestational type I/II diabetes, multiple pregnancy, and miscarriage before 20 weeks of gestation were excluded. Ethical approval was waived by the Institutional Review Board as the study was classified as a hospital audit of current clinical practice. We performed our analysis in accordance with the Helsinki Declaration of 1975 and respecting pregnant privacy. All pregnant women signed a written informed consent form before undergoing any study-specific procedures.

### 2.2 Statistical analysis

Results are reported as mean  ±  SD. GraphPad Prism 9.5 software was utilized to perform *t*-test analysis. We compared fasting glucose blood concentrations with those after 60′ and 120′ OGTT among the three groups analyzed. Statistical significance was set at *P* < 0.05.

## 3 Results

**Figure 1:**
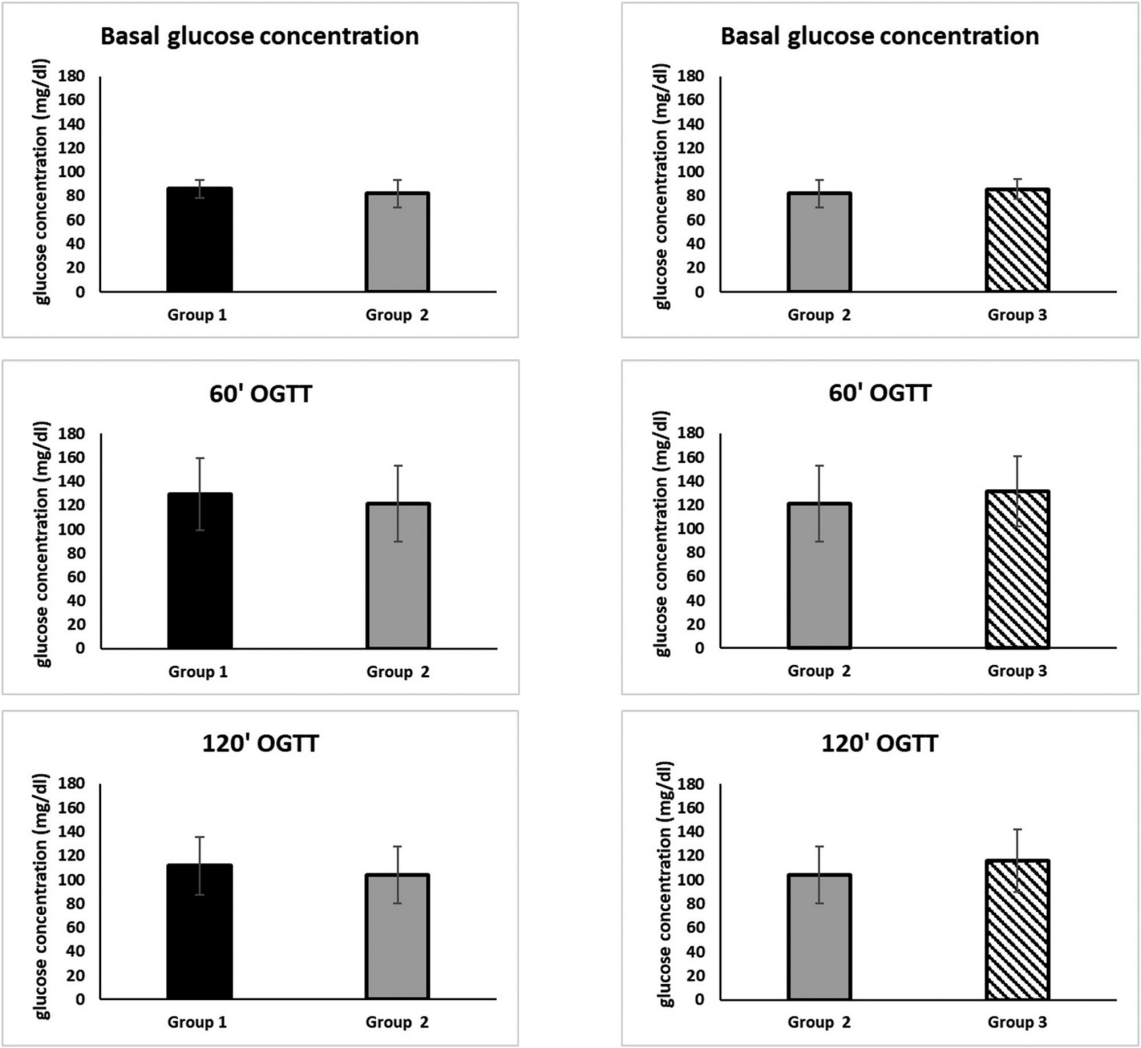
Comparison of the glucose concentration at time 0–60′–120′ between group 2 and group 1 and group 3.

As shown in Table 1 and Figure 1, the fasting glucose concentration during the COVID-19 lockdown was lower than that assessed before and after the COVID-19 lockdown. Specifically, at time 0, it was 82.14 mg/dl for group 2 vs 85.94 for group 1 (*p* = 0.0001), and 85.87 for group 3 (*p* = 0.001) (Figure 1). Similarly, the glucose concentration after 60′ OGTT for group 2 was lower than those of groups 1 and 3. In particular, at 60′, it was 121.38 mg/dl for group 2 vs 129.30 mg/dl for group 1 (*p* = 0.0029), and 131.68 mg/dl for group 3 (*p* = 0.0006) (Figure 1). Moreover, glycemia at 120’ OGTT during the COVID-19 lockdown was lower than that assessed before and after the COVID-19 lockdown. At 120′, it was 104.20 mg/dl for group 2, vs 111.51 mg/dl (*p* = 0.0004) for group 1, and 116.06 mg/dl for group 3 (*p* = 0.0001) (Figure 1).

**Table 1:** Mean and standard deviation of glucose concentrations at different time points in the three groups analyzed

		Group 1	Group 2	Group 3
		*N* (435)	*N* (193)	*N* (234)
Basal glucose concentration	Mean	85.94	82.14	85.87
	SD	7.22	11.65	8.42
60′ OGTT	Mean	129.30	121.38	131.70
	SD	30.20	31.75	29.41
120′ OGTT	Mean	111.51	104.20	116.07
	SD	24.02	23.80	25.60

## 4 Discussion

During the COVID-19 pandemic, people experienced an unknown and deadly viral infection that represented a risk for pregnant women and fetuses [21]. The COVID-19 pandemic has considerably challenged the healthcare system worldwide, resulting in the impossibility to timely providing an effective diagnosis and treatment. Thus, the Italian Diabetes and Pregnancy Study Group and a group of experts developed recommendations to guide physicians in hyperglycemia management during pregnancy [22]. COVID-19 lockdown forced changes in behavior and lifestyle in pregnant women. In addition, the COVID-19 pandemic has raised not only economic stress but also emotional stress, which notoriously increases glycemia [7,8,9]. The present study compared the GDM screening test results obtained by OGTT in 862 pregnant women aged between 17 and 47 years during, before, and after the COVID-19 lockdown. A statistically significant decrease in blood glucose concentration was observed during the lockdown compared to the previous and following years. Thus, it seems that the stressful lockdown exerted an unexpected positive effect on glycemia during pregnancy. Current studies have shown inconsistent results regarding the correlation between glycemia and COVID-19 lockdown, with some reporting an augmented blood glucose concentration in pregnant women and supporting an increase in GDM risk, while others have highlighted no differences. This discrepancy could be related to study population heterogeneity and GDM testing recommendations [23,24,25,26,27,28]. A retrospective study conducted in Lille, France, compared fasting blood glucose and postprandial blood glucose concentrations in pregnant women during the COVID-19 lockdown (222 patients enrolled in 2020) and the previous year (229 patients enrolled in 2019). The blood postprandial glucose concentration was significantly less controlled in 2020, and insulin use was significantly higher in 2020 than in 2019 (47.7 and 36.2%, respectively). To explain this evidence, it has been proposed that during the COVID-19 pandemic, physical activity rate reduction and incorrect food habits have been played out [13]. Another single-center retrospective study conducted between June 2019 and December 2020 enrolled 1,295 patients with low-risk pregnancies before the COVID-19 lockdown (June 2019 to March 2020) and during the lockdown (March 2020 to December 2020). The GDM incidence was significantly higher during the lockdown period than before the pandemic [23]. Similarly, a study conducted between January 2017 and December 2021 at the Municipal Emergency Hospital of Timisoara, Romania, revealed that GDM diagnosis was significantly increased during the COVID-19 period compared to the pre-COVID-19 one [29]. In addition, quarantine aggravated GDM pregnant women conditions and induced more adverse pregnancy outcomes during the COVID-19 outbreak [30].

Some surveys have reported no differences in the eating and sporting habits of pregnant women during the pandemic. Indeed, a survey involving 706 pregnant women in the United States conducted in May 2020 to evaluate self-reported changes in diet, physical activity, and sleep habits during the COVID-19 pandemic revealed that about 17% of women reported that their diet had worsened during the COVID-19 pandemic, 42% reported improvements, and 41% reported no change. Regarding physical activity, 22% reported a sedentary onset, 2% reported an active lifestyle, and 76% reported no change. About one-third of the participants reported a reduction in sleep hours [11]. Another survey involving 82 pregnant women with diabetes living in New Zealand revealed no differences in diet during the lockdown; thus, diabetes was not compromised during the lockdown (May 2020) [31]. During the COVID-19 lockdown, pregnant women experienced fear of contagion and limited access to physician care, triggering great control over their diet and lifestyle. Therefore, we can hypothesize that our population has not worsened its diet and physical activity, and instead, the high available free time has been employed by practicing sports and following a healthy and balanced diet. However, data on diet, physical activity, and validated scales to assess perceived stress and anxiety are unavailable. Consequently, the effects of these factors have not been evaluated.

A major strength of our study is that it included a large cohort of pregnant women. The large sample size provided adequate statistical power for the analysis. In addition, we performed a single-center analysis in which healthcare changes induced by the COVID-19 pandemic were consistent. However, our study has some limitations. First, it is a retrospective analysis that lacks standardized stress records or questionnaires to obtain information about economic/social status and the perception of individual lockdown by pregnant women. Furthermore, we have no information regarding the family context, social support, or cultural and ethnic background of the pregnant women. Moreover, there is no information on depression or food intake. Thus, the study design did not allow for the analysis of correlations among stress factors, behavior, lifestyle, and GDM in pregnancy. In addition, it has been performed in a single-center setting and with a short time frame. Moreover, our analysis did not account for the specific exposure timing and duration for each pregnancy.

## 5 Conclusion

Here, we report that the blood glucose concentration of pregnant women assessed by OGTT was lower during the COVID-19 lockdown than during the previous and following years. The findings of our study are in contrast with observational evidence of an increased risk of GDM related to the COVID-19 pandemic. Further studies are needed to better clarify the influence of lockdown restrictions on glucose metabolism and, consequently, on GDM risk, which is critical to maternal and fetal health.
